# The Influence of EGCG on the Pharmacokinetics and Pharmacodynamics of Bisoprolol and a New Method for Simultaneous Determination of EGCG and Bisoprolol in Rat Plasma

**DOI:** 10.3389/fnut.2022.907986

**Published:** 2022-05-31

**Authors:** Weiwei Zeng, Sixian Lao, Yi Guo, Yufeng Wu, Min Huang, Brian Tomlinson, Guoping Zhong

**Affiliations:** ^1^The Second People's Hospital of Longgang District, Shenzhen, China; ^2^Shenzhen Baoan Women's and Children's Hospital, Jinan University, Shenzhen, China; ^3^School of Pharmaceutical Sciences, Institute of Clinical Pharmacology, Sun Yat-sen University, Guangzhou, China; ^4^Guangdong Provincial Key Laboratory of New Drug Design and Evaluation, Guangzhou, China; ^5^Faculty of Medicine, Macau University of Science and Technology, Macau, China

**Keywords:** bisoprolol, epigallocatechin-3-gallate (EGCG), green tea, pharmacokinetics, pharmacodynamics, hypertension

## Abstract

**Background and Aim:**

Research has shown that green tea catechins may influence the activity of drug metabolizing enzymes and drug transporters. We examined whether epigallocatechin-3-gallate (EGCG) affected the pharmacokinetics and pharmacodynamics of bisoprolol in rats.

**Methods:**

A sensitive, specific liquid chromatography-tandem mass spectrometry (LC-MS/MS) method was established for the quantitative determination of EGCG and bisoprolol. The pharmacokinetic parameters of EGCG and bisoprolol in Sprague-Dawley (SD) rats were analyzed using non-compartmental methods with the aid of the computer program WinNolin. Blood pressure (BP) of spontaneously hypertensive rats (SHRs) was monitored by the tail-cuff method. Bisoprolol was given as single doses of 10 mg/kg with or without EGCG 100 mg/kg by gavage or by intravenous injection.

**Results:**

Intake of EGCG with bisoprolol by gavage significantly reduced the C_max_ (mean C_max_ from 2012.31 to 942.26 ng/mL, *P* < 0.05) and increased the T_max_ (mean T_max_ from 0.5 to 0.83 h, *P* < 0.01) for bisoprolol. After intravenous injection, EGCG significantly increased the apparent volume of distribution of bisoprolol (mean Vz/F from 1629.62 to 2473.27 mL/Kg, *P* < 0.05) and tended to increase the clearance. The absolute bioavailability of bisoprolol was reduced from 92.04 to 66.05% in rats when bisoprolol was administered with EGCG. Heart rate reduction was less in SHRs when EGCG was given by gavage with bisoprolol whereas BP reduction occurred more rapidly.

**Conclusion:**

This study showed that the simultaneous administration of EGCG by gavage at a dose of 100 mg/kg was associated with decreased C_max_ and increased T_max_ of bisoprolol, and the Vz/F of bisoprolol was increased when administered with EGCG by intravenous injection in SD rats. Moreover, the early heart rate reduction with bisoprolol was attenuated and BP reduction occurred earlier when EGCG was given with bisoprolol by gavage in SHRs.

## Introduction

The behavior of drinking tea has a history of more than 5,000 years and tea has become the second most consumed beverage in the world after water with total annual sales exceeding $43 billion globally, of which more than $11 billion is accounted for by green tea (1). In Asian countries, the average green tea consumption is about three cups per day, providing 240–320 mg of polyphenols (2). Epigallocatechin-3-gallate (EGCG) is the main abundant catechin in green tea, accounting for 50–80% of the total catechins (3). EGCG is a potent antioxidant and is considered to have the potential to treat various human diseases such as cancer, inflammation, endometriosis, diabetes, cardiovascular disease and even has antiviral activity against severe acute respiratory syndrome coronavirus-2 (SARS-CoV-2) infection (4–7).

Because of these benefits, green tea is often taken concomitantly with therapeutic drugs in many conditions, which leads to the potential for herb–drug interactions (HDIs). The major pathways responsible for HDIs involve the inhibition or induction of cytochrome P450 (CYP) enzyme activity and the expression of drug transporters such as P-glycoprotein (P-gp) and organic anion transporting polypeptide 1A2 (OATP1A2).

Bisoprolol is a β1-blocker with high oral bioavailability and is one of the first choices for the treatment of hypertension and angina. The highly β1-selective property of β-blockers has the advantage of reducing side effects and improving efficacy in the treatment of hypertension and other cardiovascular diseases (8). Bisoprolol is moderately lipophilic with an oral bioavailability of more than 90% and its half-life is 10–12 h (9). It is used once a day for the treatment of hypertension. Bisoprolol is rapidly absorbed after oral administration, and reaches peak plasma concentration after about 3 h and is eliminated with 50% renal excretion as unchanged drug and 50% *via* hepatic metabolism to pharmacologically inactive metabolites which are then excreted by the kidneys (10). Bisoprolol is mainly metabolized by the drug metabolizing enzymes CYP3A4 (95%) and CYP2D6 (5%) both of which are isoenzymes of cytochrome P450 (CYP). Therefore any drugs or herbs that induce the activity of CYP3A4 and CYP2D6 can accelerate the metabolic clearance of bisoprolol and result in drug interactions. Furthermore, the absorption and excretion of bisoprolol is closely related to the activity of OATP1A2 and P-gP (11). Therefore, any concomitant drug that affects the activity of CYP3A4, CYP2D6, P-gP or OATP1A2 may lead to changes in the plasma concentration of bisoprolol, which may be the molecular basis for the pharmacokinetic interactions between other drugs or herbs and bisoprolol.

In recent years, many studies have reported the influence of green tea extract and its major ingredient, EGCG, on drug interactions, which ultimately affect the blood concentration and efficacy of the drug. Therefore, the present study was performed to investigate the effect of EGCG on the pharmacokinetics and pharmacodynamics of bisoprolol in rats.

## Materials and Methods

### Chemicals and Reagents

EGCG (purity > 99%) and bisoprolol fumarate (purity > 99%) were purchased from Selleckchem, USA. The Bisoprolol-D7 isotope (internal standard [IS], HPLC grade) and loratadine (IS of EGCG) were obtained from CDN isotopes, Canada. LC–MS grade acetonitrile and methanol were purchased from TEDIA, USA. Ethylenediaminetetraacetic acid (EDTA) and Vitamin C were obtained from Source Leaf Organisms, China. Other chemicals employed throughout the experiment were of analytical grade and available commercially. Deionized and double-distilled water used in all assays was produced by a Milli-Q purification System (Millipore, Bedford, MA).

### Apparatus and Analytical Conditions

Samples were analyzed through Dionex Ultra-Liquid Chromatography (Thermo ScientificTM, USA) and TSQ QuantumTM Access MAX (Thermo ScientificTM, USA) systems on a Hypurity C18 column (50 × 2.1 mm,3 μm) using an injection volume of 10 μL. The mobile phases consisted of 0.1% formic acid water (A) and acetonitrile (B). The gradient elution system was optimized as follows: 0–0.5 min 10% B, 0.5–0.8 min 10–95% B, 2.0–2.2 min 95–10% B, 2.2–2.5 min 10% B. The flow rate was 0.8 mL/min. The autosampler was maintained at 4°C, and 100 μL was automatically injected into the system. MS/MS data acquisition was performed under negative/ positive electrospray ionization (ESI) mode. The Multiple Reaction Monitoring (MRM) mode was employed to monitor EGCG, bisoprolol, loratadine (IS) and D7-Bisoprolol (IS) with the precursor-to-product ion transition of m/z 457.13 → 169.98, m/z 325.96 → 115.96, m/z 383.01 → 336.94 and m/z 325.96 → 115.96, respectively. The parameters were optimized as follows: a capillary voltage of −3500 V, a gas flow rate of 12 L/min, and the dry gas temperature of 350 °C. For EGCG, bisoprolol, loratadine (IS) and D7-Bisoprolol (IS), the collision energies were 20 eV, 17 eV, 18 eV, and 24 eV, respectively. The other parameters, including cone voltage (CV), collision energy (CE), and the dwell time, were also achieved for the maximum abundance of the ions of interest by the automatic tune procedure of the instrument. Thermo Xcalibur (2.2 SP1.48, Thermo Scientific, USA).

### Preparation of Calibrators and Quality Control (QC) Samples

An 8-level series of calibrators was prepared using pooled plasma. Briefly, pooled plasma was spiked with working solution to give 50,100,500,1,000,2,000,5,000,10,000 and 20,000 ng/mL calibrators. Pooled plasma was used as blank. Calibrators were aliquotted and stored at −80°C. A 4-level QC was prepared using pooled plasma. Briefly, pooled plasma was spiked with working solution to give 15,000,1500,150 and 50 ng/mL QC. All QC samples were aliquotted and stored at −80°C.

### Extraction Procedure

50 μL of Plasma,d7-Bisoprolol (1 μg/mL),Loratadine (1 μg/mL) and Antioxidant Mixture Were Added Into a 1.5 mL Centrifuge Tube, After 1 min of Vortex Mixing, 200 μL of Acetonitrile Was Added and Vortex Mixed for 3 min, Then Mixed Evenly and let to Stand at Room Temperature for 5 min. After 5 min of Centrifugation at 1400 rpm, 180 μL of Supernatant Was Added Into Another Clean EP Tube and Centrifuged Again at 14,00 rpm for 5 min, Then 10 μL of the Supernatant Was Taken and Subjected to LC-MS/MS Analysis.

### Bisoprolol-EGCG Pharmacokinetic Interaction

A total of 30 Sprague-Dawley (SD) rats including 15 male and 15 female, weighing 180–220 g, were acquired from the Laboratory Animal Center of Sun Yat-Sen University (Guangzhou, China, license no. SCXK 2016-0029). The SD rats were housed in clean cages under an optimal temperature range of 24–26°C and 12 h light/dark cycle with free access to food and water. All the animal procedures complied with the institutional animal ethics guidelines set by the Animal Care and Use Committee of Sun Yat-Sen University. The rats were randomly allocated to two phases of this pharmacokinetic interaction study. The dosing of 18 rats was given by intragastric (i.g.) gavage and another 12 rats were dosed by tail vein injection. The 18 rats were randomly allocated to three groups: bisoprolol (10 mg/kg) group, EGCG (100 mg/kg) group and the bisoprolol (10 mg/kg) + EGCG (100 mg/kg) combination group. The 12 rats were randomly allocated to two groups: bisoprolol (10 mg/kg body weight, i.g.) group, bisoprolol (10 mg/kg body weight, i.g.) + EGCG (100 mg/kg body weight, i.g.) group. The dose of the 12 rats was calculated from bisoprolol (10 mg/kg) and EGCG (100 mg/kg) dissolved in a 0.7% saline solution. The rats were fasted for 12 h prior to drug administration without restriction of drinking water, and feeding restarted at 4 h after dosing. The blood samples (approximately 0.3 mL) were collected into heparinized centrifuge tubes *via* orbital venous plexus sampling before dosing (denoted as 0 min), and at 5 min,10 min,15 min,30 min,1 h,1.5 h,2 h,4 h,8 h, and 24 h after dosing. The supernatant was collected by centrifugation of the blood samples immediately at 4000 rpm for 10 min at 4°C and stored at −20°C until further analysis.

### Blood Pressure Measurements of SHRs

Twelve male spontaneously hypertensive rats (SHRs) of body weight about 320 g and average blood pressure more than 180 mm Hg were used. The SHRs were divided into two groups and 6 in each group (including three female and three male). The rats were housed in clean cages under an optimal temperature range of 24–26°C and 12 h light/dark cycle with free access to food and water. The rats were randomly allocated to the bisoprolol (10 mg/kg body weight, i.g.) group and the bisoprolol (10 mg/kg body weight, i.g.) + EGCG (100 mg/kg body weight, i.g.) group. The dose selection mainly was refered to the previous study in SHRs (12). Fasting was carried out for 12 h prior to drug administration without the restriction of drinking water, and feeding restarted at 4 h after dosing. The dosing was given by gavage. A real-time blood pressure monitor (Intelligent non-invasive blood pressure monitor mouse BP-2010A, China) with non-invasive manometry was used to measure blood pressure at 0 min,15 min,30 min,45 min,1 h,75 min,1.5 h,105 min,2 h,4 h,6 h, 8 h,12 h, and 24 h after dosing. The average value of three recordings of blood pressure from the tail artery in the awake state of the rats was used for analysis.

### Isothermal Titration Calorimetry Assay

Isothermal titration calorimetry (ITC) assay was performed to investigate the binding of EGCG to bisoprolol on a NanoITC LV-190 μL (Waters GmbH, TA Instruments, Eschborn, Germany). Titration calorimetry was performed at 25°C in the assay buffer (PBS, PH 6.5). Briefly, the sample and syringe cell were filled with bisoprolol (2.1 mM) and EGCG (13 mM), respectively, which were degassed prior to use. The titrations consisted of 25 consecutive injections of 1.96 μL each with a 200 s interval between injections. The data were analyzed using the instrumental internal software package and fitted with an independent model.

### Ethics Statement

All animal studies were carried out in strict accordance with the recommendations in the Guide for the Care and Use of Laboratory Animals of the Laboratory Animal Center of Sun Yat-Sen University. The protocol was approved by the Institutional Review Board of Baoan Women's and Children's Hospital, Shenzhen, China with IRB No LLSC2020-03-05, and the Animal Care and Use Committee of Sun Yat-Sen University.

### Pharmacokinetic Analysis

The pharmacokinetic parameters of bisoprolol were calculated using non-compartmental methods with the aid of the computer program WinNolin (version 8.1, Pharsight Corporation). C_max_ and T_max_ were obtained directly from the observed plasma concentration-time data. The terminal elimination rate constant (λ_Z_) was determined by linear regression of the terminal portion of the plasma concentration-time curve and the elimination half-life (t_1/2_) was calculated as 0.693/λ_Z_. Systemic exposure to bisoprolol was evaluated by calculating the AUC using the linear trapezoidal rule and AUC_0−∞_ was calculated as AUC_0−∞_ = AUC_0−*t*_ + C_t_/K_el_ where C_t_ is the last quantifiable concentration. Apparent volume of distribution (Vz/F) = dose ^*^ bioavailability / plasma drug concentration. The oral clearance (CL/F) was calculated as Dose/AUC_0−∞._

### Statistical Analysis

All data were verified for normal distribution. Data are presented as mean ± SD. A probability value <0.05 was considered statistically significant. The pharmacokinetic parameters of bisoprolol with and without EGCG were compared by repeated measures ANOVA and the Friedman rank test was used to compare T_max_ values. SPSS 25.0 for Windows (SPSS, Chicago, IL) was used.

## Results

### The Direct Binding Effect of EGCG and Bisoprolol *in vitro*

**Figure 1:**
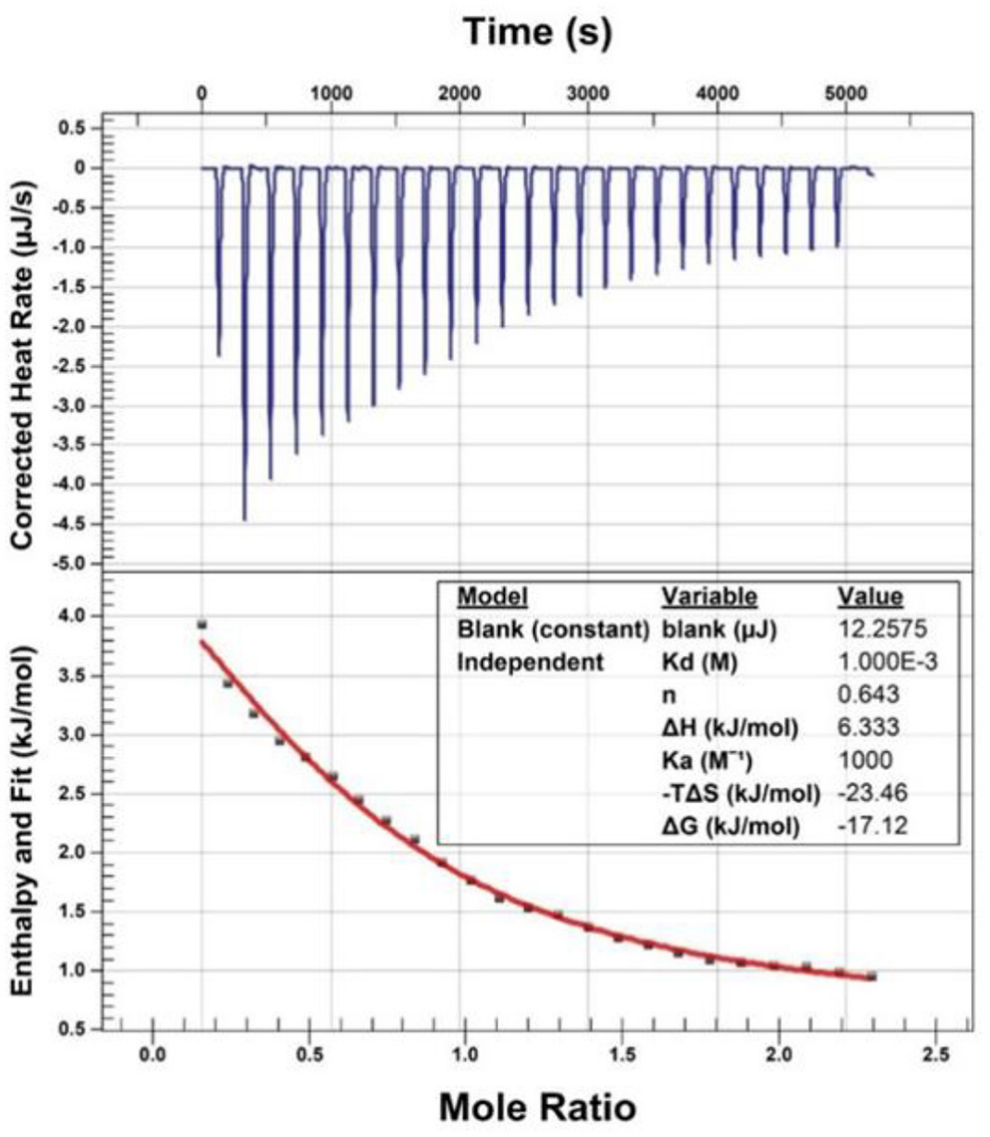
Isothermal titration calorimetry (ITC) assay.

In this assay, bisoprolol was titrated with EGCG at room temperature. The thermodynamics parameters of interaction between EGCG and bisoprolol can be calculated by fitting the raw ITC data. The parameter values were as follows: ΔGo = −17.12 kJ/mol, ΔHo = 6.33 kJ/mol, –TΔSo = −23.46 kJ/mol. The equilibrium dissociation constant (KD) was determined after analysis of the normalized ITC curve by the NanoAnalyze Software. The data indicated that EGCG can bind to bisoprolol (see Figure 1).

### Assay Validation

**Figure 2:**
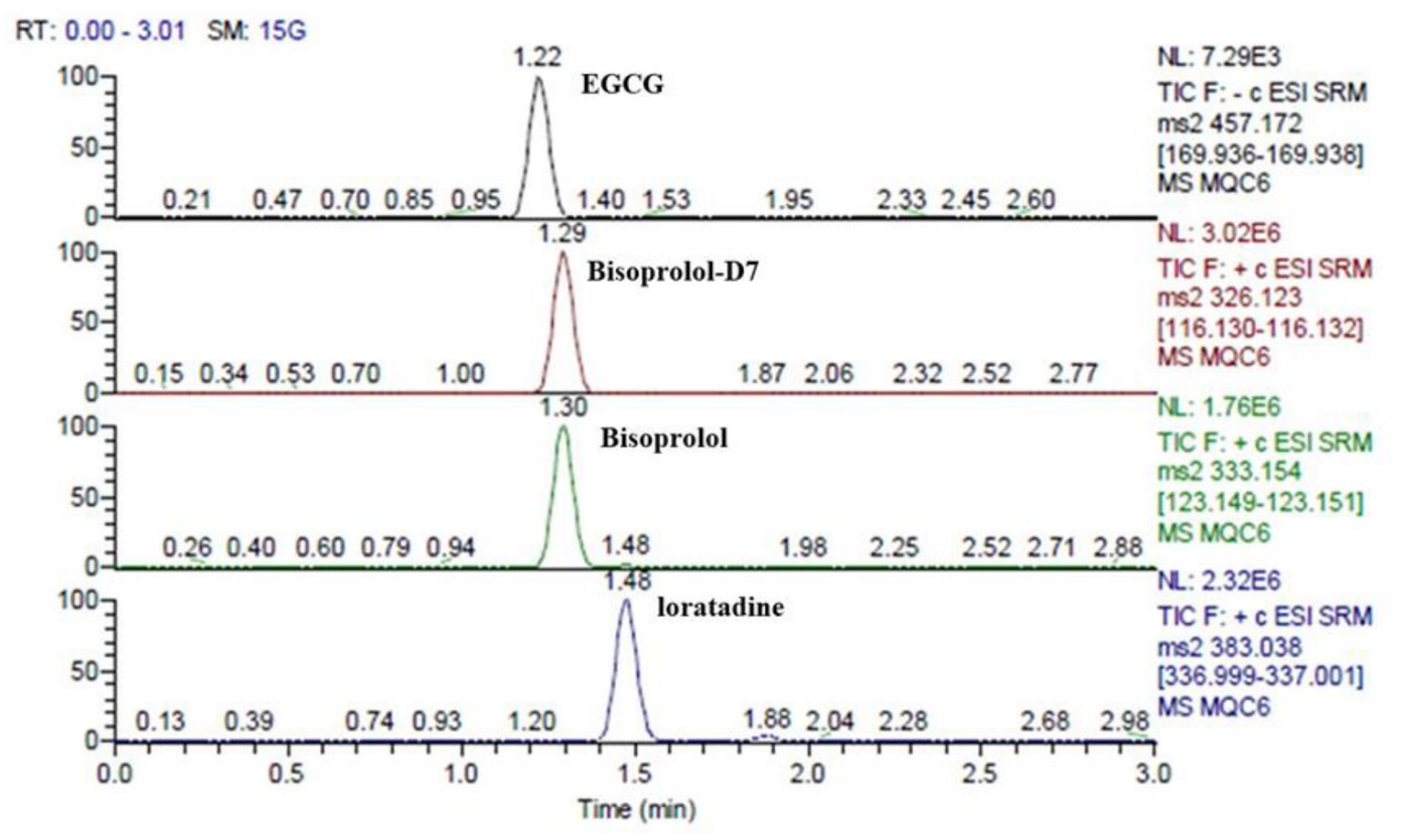
The retention times of EGCG, bisoprolol, deuterated bisoprolol and loratadine in plasma sample at the same time.

The retention times of EGCG, bisoprolol, deuterated bisoprolol and loratadine were 1.22, 1.29, 1.30, and 1.48 min, respectively. There were no impurity peaks in the blank plasma samples at or near the peak time (Figure 2). The linear relationship between EGCG and bisoprolol was present in the range of 5–2,000 ng/mL, and the correlation coefficient (R^2^) was more than 0.99. The deviation of each concentration in the standard curve was within the acceptable range of ±15%. The standard curve results are shown in Table 1. Accuracy and precision all met acceptable requirements, precision (RSD, %) were <15% and accuracy (RE, %) were within ±15%. The accuracy and precision of LLOQ did not exceed 20% (Table 2). The recoveries and matrix effects of EGCG and bisoprolol are shown in Table 3. The recoveries for all analytes ranged from 61.0 to 99.7%. No significant signal was observed in the mass spectrometry affecting rat plasma. Under current analytical conditions, matrix effects were negligible. The accuracy of bisoprolol and EGCG measurement was not significantly affected under different storage condition including 12 h in the autosampler, 4 h on a laboratory table at room temperature, 30 days in a low temperature freezer at −80°C or 3 freeze-thaw cycles (Table 4). No significant differences were observed in test results compared to freshly prepared samples.

**Table 1:** Regression equation of EGCG and bisoprolol in rats plasma determined by HPLC-MS (weight W=1/X^2^).

**Analyte**	**Calibration curves**	**R^**2**^**	**Weight**	**%RE**
EGCG	Y = 3.028e^−5^X - 1.009e^−4^	0.9982	1/X^2^	−7.37 ~ 4.19
	Y = 2.098e^−5^X - 7.199e^−5^	0.9936	1/X^2^	−13.15 ~ 9.91
	Y = 2.144e^−5^X - 7.308 e^−5^	0.9918	1/X^2^	−11.19 ~ 10.60
Bisoprolol	Y = 1.193e^−2^X + 3.506e^−3^	0.9996	1/X^2^	−3.16 ~ 1.91
	Y = 1.117e^−2^X + 1.742e^−5^	0.9991	1/X^2^	−5.00 ~ 4.25
	Y = 1.119e^−2^X + 2.248e^−3^	0.9992	1/X^2^	−4.60 ~ 3.51

**Table 2:** Intra-day and inter-day precisions and accuracies for the determination of EGCG and Bisoprolol from the assay samples (mean ± SD, *n* = 6).

	**Concentration**	**Intra batch**			**Inter batch**		
**Analyte**	**(ng/ml)**	**Mean ±SD**	**RE (%)**	**RSD (%)**	**Mean ±SD**	**RE (%)**	**RSD (%)**
EGCG	5	5.79 ± 1.06	15.77	18.35	5.99 ± 0.76	19.70	12.63
	15	15.48 ± 1.13	3.17	7.32	14.61 ± 1.10	−2.60	7.50
	150	137.34 ± 6.48	−8.44	4.72	136.75 ± 1.31	−8.84	0.96
	1500	1,547.44 ± 32.52	3.16	2.10	156.87 ± 48.11	4.19	3.08
Bisoprolol	5	4.23 ± 0.21	−15.42	4.89	4.30 ± 0.10	−13.95	2.37
	15	14.04 ± 0.31	−6.40	2.21	14.15 ± 0.51	−5.66	3.57
	150	150.30 ± 1.91	0.20	1.27	152.75 ± 2.99	1.83	1.96
	1500	1,502.17 ± 18.52	0.14	1.23	1,508.16 ± 10.97	0.54	0.73

**Table 3:** Extraction recovery and matrix effect for the EGCG and Bisoprolol in plasma (mean ± SD, *n* = 6).

	**Concentration**	**Matrix effect**		**Recovery**	
**Analyte**	**(ng/ml)**	**Mean ±SD**	**RSD (%)**	**Mean ±SD**	**RSD (%)**
EGCG	1500	90.12 ± 4.13	4.58	79.09 ± 2.97	3.76
	150	91.00 ± 4.59	5.04	60.96 ± 4.20	6.89
	15	97.58 ± 4.05	4.15	72.69 ± 10.22	14.06
Bisoprolol	1500	99.91 ± 2.14	2.14	98.82 ± 5.07	5.15
	150	98.53 ± 1.42	1.45	99.71 ± 3.49	3.50
	15	100.14 ± 3.34	3.34	96.88 ± 7.72	7.96

**Table 4:** Stability of EGCG and Bisoprolol in plasma (mean ± SD, *n* = 6).

**Storage condition**	**Analyte**	**Concentration (ng/ml)**	**Mean ±SD (ng/ml)**	**RSD (%)**	**RE (%)**
Room temperature 4 h	EGCG	15	14.13 ± 1.76	12.44	−5.80
		150	131.46 ± 15.90	12.09	−12.36
		1500	1,446.46 ± 24.61	1.70	−3.57
	Bisoprolol	15	14.06 ± 0.41	2.90	−6.36
		150	151.73 ± 7.25	4.78	1.15
		1500	1,500.19 ± 24.77	1.65	0.01
Autosampler for 12 h (4°C)	EGCG	15	14.49 ± 1.60	11.02	−3.41
		150	135.76 ± 5.73	4.22	−3.41
		1500	1,506.53 ± 49.58	3.29	0.44
	Bisoprolol	15	13.68 ± 0.33	2.41	−8.82
		150	151.72 ± 2.33	1.54	1.15
		1500	1,519.58 ± 23.01	1.51	1.31
Three Freeze/thaw cycle at −80°C	EGCG	15	13.48 ± 1.13	8.41	−10.11
		150	129.87 ± 6.29	4.48	−13.42
		1500	157.56 ± 63.32	4.02	5.04
	Bisoprolol	15	14.89 ± 0.51	3.43	−0.72
		150	150.54 ± 1.10	0.73	0.36
		1500	144.94 ± 22.43	1.50	0.01
Long term for 30 d at −80 °C	EGCG	15	12.93 ± 1.03	7.99	−13.80
		1500	1,391.63 ± 87.32	6.27	−7.22
	Bisoprolol	15	14.44 ± 0.33	2.26	−3.70
		1500	1,432.24 ± 44.86	3.13	−4.52

### Effect of EGCG on the Pharmacokinetics of Bisoprolol

**Figure 3:**
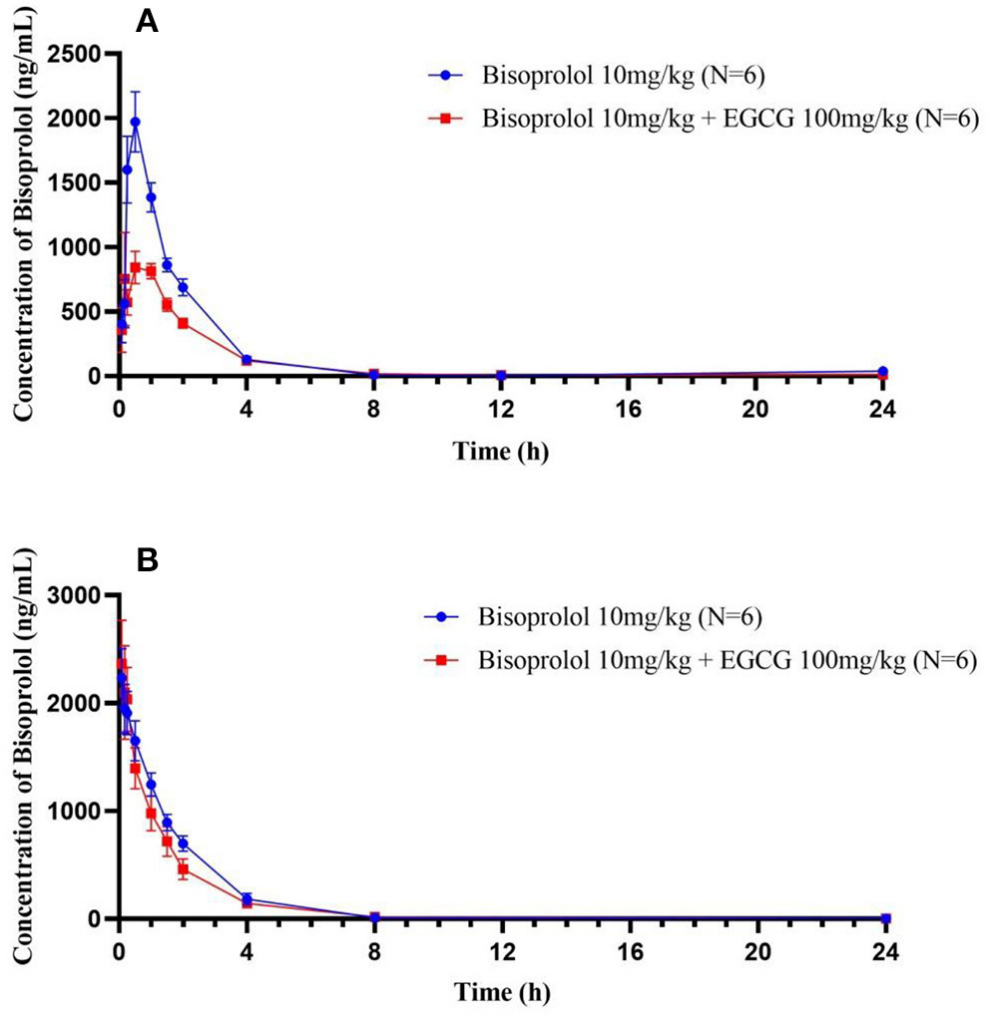
The plasma concentration time curves of bisoprolol after oral **(A)** and intravenous **(B)** administration.

After intragastric administration, intake of EGCG (100 mg/kg body weight) with bisoprolol significantly increased the T_max_ (mean T_max_ from 0.5 to 0.83 h, *P* < 0.01) and reduced the C_max_ (mean C_max_ from 2012.31 to 942.26 ng/mL, *P* < 0.05) of bisoprolol (Figure 3 and Table 5). Moreover, intake of EGCG tended to reduce the systemic exposure to bisoprolol (mean AUC_0−∞_ from 3914.09 to 2347.37 h.ng/mL, *P* = 0.17) but this was not significant and there was no significant effect on the elimination half-life (t_1/2_; Figure 3 and Table 5). After intravenous administration, intake of EGCG with bisoprolol significantly increased the apparent volume of distribution (mean *V*_z_/F from 1629.62 to 2473.27 mL/Kg, *P* < 0.05) but there was no significant effect on other pharmacokinetic parameters (Figure 3 and Table 5). In the male group, the trend of increasing Cl/F after co-administration was stronger than in the female group. This means that the inhibitory effect of EGCG on the elimination of bisoprolol was stronger in the male rats. AUC_0−*t*_ and AUC_0−∞_ also went down in the male group more than in the female group, which means that EGCG had more of an impact on bisoprolol's absorption than it did in the female rats. So, it can be said that there is a gender difference in the way EGCG affects the way bisoprolol is absorbed, used, and excreted (Data didn't shown).

**Table 5:** Effect of EGCG on the pharmacokinetic parameters of bisoprolol in SD rats.

**Parameters**	**Intragastric administration**		**Intravenous administration**	
	**Bisoprolol (*n* = 6)**	**Bisoprolol+EGCG(*n* = 6)**	**Bisoprolol (*n* = 6)**	**Bisoprolol+EGCG(*n* = 6)**
*t*_1/2_, h	3.89 ± 2.03	4.49 ± 1.45	2.47 ± 0.32	2.88 ± 0.94
*T*_max_, h	0.50 ± 0.10	0.83 ± 0.26^**^	0.25 ± 0.37	0.12 ± 0.07
*C*_max_, mg/mL	2,012.31 ± 510.67	942.26 ± 230.23^*^	2,259.56 ± 607.55	2,438.08 ± 962.08
AUC_0−*t*_, h·ng/mL	3,709.61 ± 827.46	2,260.37 ± 579.78	4,030.01 ± 1,091.01	3,421.82 ± 1,124.34
AUC_0−∞_, h·ng/mL	3,914.09 ± 1081.55	2,347.37 ± 586.96	4,050.09 ± 1,117.69	3,436.69 ± 1,115.84
*V*_z_/F, mL/Kg	3,687.71 ± 2021.28	7,693.73 ± 5,114.65	1,629.62 ± 279.05	2,473.27 ± 1,814.65^*^
Cl/F, mL/h/Kg	661.27 ± 225.56	1,028.96 ± 374.99	466.34 ± 111.29	546.72 ± 206.45

* *P <0.05*,

** * P <0.01*.

### The Pharmacokinetics of EGCG

The pharmacokinetic parameters of EGCG after intragastric administration in SD rats were shown in Table 6. There were no significant differences in a single dose of EGCG and combined with bisoprolol.

**Table 6:** Pharmacokinetic parameters of EGCG after intragastric administration in SD rats.

**Parameters**	**EGCG(*n* = 6)**	**EGCG+Bisoprolol(*n* = 6)**	***P*-value**
*t*_1/2_, h	5.06 ± 2.99	2.81 ± 0.96	0.130
*T*_max_, h	0.36 ± 0.35	0.18 ± 0.06	0.270
*C*_max_, mg/mL	6,007.28 ± 8,136.92	4,768.19 ± 4,636.59	0.753
AUC_0−*t*_, h·ng/mL	3,499.28 ± 2,489.96	3,219.20 ± 2,614.72	0.853
AUC_0−∞_, h·ng/mL	3,634.08 ± 2,400.23	3,274.21 ± 2,625.90	0.809
*V*_z_/F, mL/Kg	96,970.06 ± 109,217.92	120,821.98 ± 205,173.74	0.807
Cl/F, mL/h/Kg	11,109.16 ± 10,466.19	25,615.88 ± 39,010.30	0.400

### Effect of EGCG on the Bioavailability of Bisoprolol

Based on the pharmacokinetic study results of intragastric administration and tail vein administration, the absolute bioavailability of bisoprolol in the single-dose bisoprolol group and the combined EGCG-administered group was calculated. The results showed that the absolute bioavailability of bisoprolol when administered alone (F_abs.Bisoprolol_ = 92.04%) was greater than when it was administered in combination with EGCG (F_abs.Bisoprolo+EGCG_ = 66.05%).

### Effect of EGCG on the Pharmacodynamics of Bisoprolol

**Figure 4:**
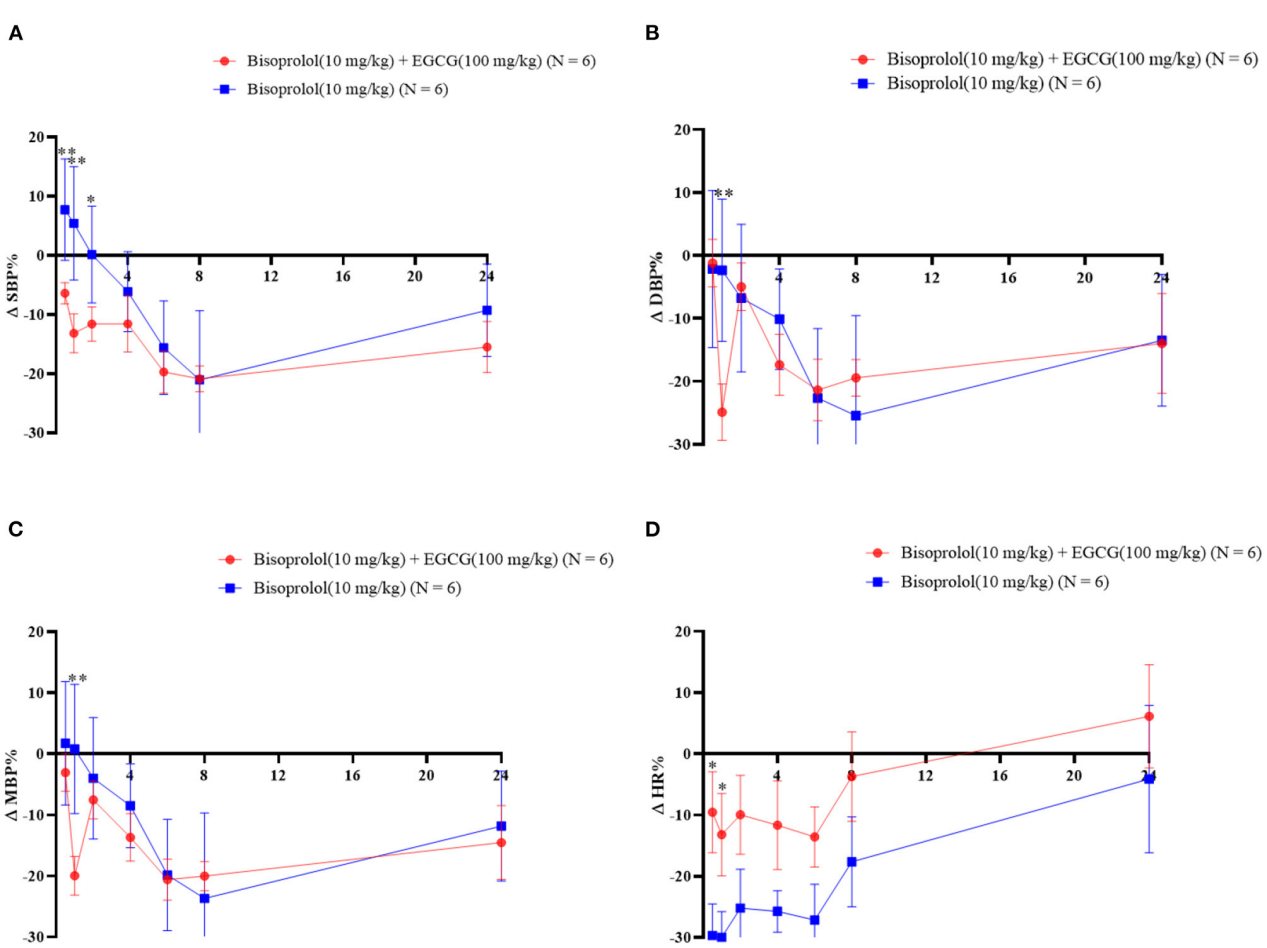
The blood pressure and heart rate changes as percentages over time after gavage administration of bisoprolol with or without EGCG. *P < 0.05, **P < 0.01. **(A)** Percentage change of SBP; **(B)** Percentage change of DBP; **(C)** Percentage change of mean BP; **(D)** Percentage change of heart rate.

After gavage administration, intake of EGCG (100 mg/kg body weight) with bisoprolol lowered SBP at the first measurement at 0.5 h post dose of EGCG and bisoprolol, while the SBP only started to decrease at 4 h after administration of bisoprolol alone. The largest reduction of SBP was 37.37 ± 10.91 mmHg (change of −20.8%) at 8 h post dose with EGCG and bisoprolol combined and was similar with bisoprolol alone. MBP and DBP reached the maximum reduction of 28.38 ± 9.30 mmHg (change of −20%) and 31.68 ± 12.96 mmHg(change of −24.6%), respectively, at 1 h post dose of EGCG and bisoprolol. However, the reduction of MBP and DBP were relatively slow, and reached the maximum reduction at 8 h after the single dose of bisoprolol. The largest reduction of heart rate (HR) was 140.72 ± 26.28 beats/min (change of −30.0%) at 1 h after the dose of bisoprolol alone and it was greater than the effect of the combination of EGCG and bisoprolol with a reduction of 60.18 ± 80.37 beats/min (change of −13.2%; Table 7 and Figure 4).

**Table 7:** The reduction of blood pressure and heart rate after treatment in the two groups in SHRs.

**Time post dose**	**SBP, mmHg**		**DBP, mmHg**		**MBP, mmHg**		**HR,beats/min**	
	**Bisoprolol**	**Bisoprolol + EGCG**	**Bisoprolol**	**Bisoprolol + EGCG**	**Bisoprolol**	**Bisoprolol + EGCG**	**Bisoprolol**	**Bisoprolol + EGCG**
0.5 h	−13.62 ± 15.41	11.72 ± 8.37^**^	3 ± 16.77	1.93 ± 11.02	−2.5 ± 14.97	4.85 ± 10.26	139.48 ± 31.73	45.28 ± 71.11^**^
1 h	−9.18 ± 16.61	23.23 ± 13.29^**^	3.68 ± 15.03	31.68 ± 12.96^**^	−0.73 ± 15.43	28.38 ± 9.30^**^	140.72 ± 26.28	60.18 ± 80.37^**^
2 h	0.17 ± 13.95	20.5 ± 12.07^**^	9.45 ± 15.97	6.17 ± 11.73	6.28 ± 14.67	10.62 ± 10.67	118.88 ± 36.24	47 ± 74.06
4 h	10.92 ± 11.59	21.05 ± 21.14	14.22 ± 11.12	23.07 ± 16.13	13.05 ± 10.23	20.43 ± 15.31	120.78 ± 21.76	53.77 ± 79.17
8 h	36.13 ± 17.46	37.37 ± 10.91	33.78 ± 18.89	24.83 ± 9.03	34.5 ± 17.88	28.88 ± 8.27	82.78 ± 34.10	23.98 ± 80.04
24 h	16.08 ± 13.65	26.83 ± 18.43	18.48 ± 14.05	18.6 ± 26.53	17.7 ± 13.32	21 ± 22.24	20.08 ± 54.12	−21.43 ± 71.55

*Negative values indicate increases*.

* *P <0.05*,

** *P <0.01*.

## Discussion

Green tea is taken as a common drink worldwide and it can interact with various medications and may alter their pharmacokinetic and pharmacodynamic properties. In this study, we found for the first time that the green tea component EGCG (100 mg/kg) can reduce the C_max_ of bisoprolol and delay the T_max_ and tend to reduce the AUC when given simultaneously by gavage in rats, while the Vz/F and clearance of bisoprolol tended to increase. The T_max_, C_max_ and the AUC are all related to the absorption process of bisoprolol. The results suggest that EGCG can inhibit the absorption of bisoprolol when given together and this can lead to a lower early reduction in HR. The attenuation in reduction of HR with EGCG combined with bisoprolol was associated with a greater early reduction in SBP, which may reflect the various mechanisms involved in the reduction of BP with a beta-blocker. In order to detect the plasma concentrations of bisoprolol and EGCG simultaneously, a new HPLC-MS/MS method was developed and validated. The method meets the guidelines for the validation of methods for the definitive analysis of biological samples (Version 2020) and was applied for the detection of bisoprolol and EGCG in this study. We also employed isothermal titration calorimetry (ITC), a thermodynamic method with high sensitivity and reproducibility *in vitro*, to study biomolecular interactions. We tested the direct binding effect of EGCG and bisoprolol *in vitro* and found that EGCG and bisoprolol showed a significant binding phenomenon, similar to that of EGCG and atenolol.

EGCG is the main abundant catechin in green tea, accounting for 50–80% of the total catechins and has been reported to improve many cardiovascular risk factors including blood pressure (13). Intake of catechins 400–500 mg daily can significantly reduce systolic and diastolic blood pressure (14). However, several studies have reported that green tea polyphenols may affect the expression or activities of drug-metabolizing enzymes such as CYP1A1, CYP2D6, CYP3A4 and drug transporters which leads to changes in the absorption and metabolism of certain drugs (15, 16). In addition, it was reported that green tea catechins can inhibit the activities of CYP1A1, CYP2A6, CYP2C9 and CYP3A4 (17). Some other studies have also reported the inhibition of the ABCB1 and ABCG2 transporter activity by EGCG (18). Roth et al. showed that EGCG inhibited OATP1A2— and OATP2B1-mediated uptake of estrone-3-sulfate in an *in vivo* study (19). Another study has shown that the consumption of green tea extract can significantly reduce the bioavailability of nadolol through the inhibition of OATP1A2-mediated uptake (20). Therefore, EGCG may inhibit the efflux of bisoprolol by inhibiting the action of P-gp, resulting in increased bioavailability.

A study in a Korean population found that co-administration of rosuvastatin with EGCG resulted in a 19% decrease in the plasma concentrations of the drug apparently due to inhibition of drug absorption by the transporters OATP1A2 and OATP2B1(21). In a study in Hong Kong, green tea extract was reported to affect the pharmacokinetics of rosuvastatin and reduced the peak plasma drug concentration by 30% (22). It has also been reported that green tea was able to inhibit the drug metabolizing enzyme CYP3A4 and the efflux transporter P-gP for simvastatin, resulting in increased simvastatin plasma concentrations (23). Moreover, in a study in Japan there was a significant effect of green tea on the pharmacokinetics and therapeutic efficacy of nadolol after 14 days of simultaneous administration of nadolol and green tea. In 10 healthy volunteers, the peak plasma concentration (C_max_) and area under the plasma concentration time curve up to 48 h (AUC_0−48_) decreased by 85.3 and 85.0%, respectively, while the time to C_max_ (T_max_) was significantly shortened, but no effect on the clearance was found. Further study identified the mechanism involved in the inhibition of the absorption of nadolol by EGCG through the transporter OATP1A2 which resulted in a decrease in the plasma concentration of nadolol (24). In addition, EGCG can interact with atenolol and form a precipitate under acidic conditions *in vitro*, resulting in the inhibition of the absorption of atenolol (25).

EGCG has been reported to increase the AUC of tamoxifen and diltiazem when combined with these drugs by inhibiting the activity of CYP3A4 and P-gp (18). Tamoxifen and diltiazem are known to be metabolized by the same pathway as bisoprolol. However, the AUC and C_max_ of bisoprolol were reduced in this study. That is mainly because of the differences in the first-pass effect during the absorption of drugs. The first-pass effect of bisoprolol is much smaller than that of tamoxifen or diltiazem. Therefore, we will further investigate the absorption pathway of bisoprolol and the factors affecting this.

The metabolism of bisoprolol is mainly dependent on the CYP3A4 enzyme, and it has been shown that EGCG can inhibit the CYP3A4 enzyme (26), so it may be predicted that EGCG could inhibit part of the metabolism of bisoprolol by inhibiting the activity of CYP3A4, resulting in a reduction in the metabolism of bisoprolol and causing accumulation. In addition, when the metabolic pathway of bisoprolol *via* CYP3A4 enzymes is inhibited, renal clearance becomes the main route of elimination of bisoprolol, so renal clearance increases and the overall elimination half-life is prolonged although no significant effect was observed in the present study.

The absorption of bisoprolol in the intestine may be dependent on organic cation transporters (OCTs) for completion (27). However, it has been shown that EGCG inhibits the activity some of the OCTs (28), so EGCG may affect the absorption of bisoprolol by inhibiting the action of OCTs, resulting in a reduction in the absorption and a delay in absorption time, leading to a delay in the T_max_ and a reduction of C_max_ and AUC. The increase in the apparent volume of distribution of bisoprolol after intravenous administration with EGCG may also be related to effects on transporters resulting in increased distribution of bisoprolol to body tissues or more rapid clearance of bisoprolol by metabolism or renal excretion.

## Limitation

This study has some limitations that need to be considered. Firstly, this study only assessed one dosage for EGCG (100 mg/kg). It is known that interactions between herbs and drugs may be dose-dependent. Evaluating a higher dose or a lower dose may help to provide a better understanding of the interaction between bisoprolol and green tea. Secondly, it has been shown that taking EGCG 8 or 4 h before sunitinib administration had no effect on the pharmacokinetics of sunitinib in rats, whereas taking the two together reduced the bioavailability of sunitinib, probably because of a physical reaction between the two compounds (50), suggesting separation of dosing of green tea and drugs may reduce any herb-drug interaction. EGCG was given simultaneously with bisoprolol in the present study and we did demonstrate by isothermal titration calorimetry that a physico-chemical reaction does occur between EGCG and bisoprolol and this may be responsible for some of the interaction. It would be useful to assess whether the separation of dosing or repeated dosing of EGCG and bisoprolol have different effects.

## Conclusion

This study showed that administration of EGCG at a single dose of 100 mg/kg with a single dose of bisoprolol of 10 mg/kg was associated with decreased C_max_ and T_max_ and a tendency for decreased AUC and increased Vz/F and clearance for bisoprolol in SD rats when bisoprolol was taken simultaneously with EGCG. Moreover, administration of EGCG significantly attenuated the early HR reduction with bisoprolol and resulted in an earlier reduction in BP compared to when bisoprolol was given alone in SHRs.

## Data Availability Statement

The original contributions presented in the study are included in the article/supplementary material, further inquiries can be directed to the corresponding authors.

## Ethics Statement

The animal study was reviewed and approved by the Institutional Review Board of Baoan Women's and Children's Hospital with IRB No LLSC2020-03-05 and the Animal Care and Use Committee of Sun Yat-sen University.

## Author Contributions

WZ and SL analyzed the data and wrote this manuscript. WZ and GZ designed the research project. SL, YG, and YW performed the experiments. MH, BT, GZ, and WZ revised this manuscript. All authors contributed to the article and approved the submitted version.

## Funding

This work was supported by National Natural Science Foundation of China [No. 82173776], Natural Science Foundation of Guangdong Province [No. 2021A1515010574], Guangdong Provincial Key Laboratory of Construction Foundation [No. 2020B1212060034], and National Key Research and Development Program [No. 2020ZX09201-021].

## Conflict of Interest

The authors declare that the research was conducted in the absence of any commercial or financial relationships that could be construed as a potential conflict of interest.

## Publisher's Note

All claims expressed in this article are solely those of the authors and do not necessarily represent those of their affiliated organizations, or those of the publisher, the editors and the reviewers. Any product that may be evaluated in this article, or claim that may be made by its manufacturer, is not guaranteed or endorsed by the publisher.
